# Rosmarinic acid exhibits broad anti-enterovirus A71 activity by inhibiting the interaction between the five-fold axis of capsid VP1 and cognate sulfated receptors

**DOI:** 10.1080/22221751.2020.1767512

**Published:** 2020-06-04

**Authors:** Chung-Fan Hsieh, Jia-Rong Jheng, Guan-Hua Lin, Yu-Li Chen, Jin-Yuan Ho, Chien-Jou Liu, Kuei-Yang Hsu, Yuan-Siao Chen, Yoke Fun Chan, Hui-Ming Yu, Pei-Wen Hsieh, Jyh-Haur Chern, Jim-Tong Horng

**Affiliations:** a Department of Biochemistry and Molecular Biology, College of Medicine, Chang Gung University, Taoyuan, Taiwan; b Genomics Research Center, Academia Sinica, Taipei, Taiwan; c Department of Medical Microbiology, University Malaya, Kuala Lumpur, Malaysia; d Graduate Institute of Natural Products, College of Medicine, Chang Gung University, Taoyuan, Taiwan; e Research Center for Industry of Human Ecology and Graduate Institute of Health Industry Technology, Chang Gung University of Science and Technology, Taoyuan, Taiwan; f Department of Anesthesiology, Chang Gung Memorial Hospital, Taoyuan, Taiwan; g Institute of Biotechnology and Pharmaceutical Research, National Health Research Institutes, Zhunan, Taiwan, ROC; h Research Center for Emerging Viral Infections and Healthy Aging Research Center, College of Medicine, Chang Gung University, Taoyuan, Taiwan; i Molecular Infectious Disease Research Center, Chang Gung Memorial Hospital, Chang Gung University College of Medicine, Taoyuan, Taiwan

**Keywords:** Enterovirus A71, five-fold axis, heparan sulfate, P-selectin glycoprotein ligand-1, receptor, rosmarinic acid, scavenger receptor B2, viral entry

## Abstract

Enterovirus A71 (EV-A71), a positive-stranded RNA virus of the Picornaviridae family, may cause neurological complications or fatality in children. We examined specific factors responsible for this virulence using a chemical genetics approach. Known compounds from an anti-EV-A71 herbal medicine, *Salvia miltiorrhiza* (Danshen), were screened for anti-EV-A71. We identified a natural product, rosmarinic acid (RA), as a potential inhibitor of EV-A71 by cell-based antiviral assay and *in vivo* mouse model. Results also show that RA may affect the early stage of viral infection and may target viral particles directly, thereby interfering with virus-P-selectin glycoprotein ligand-1 (PSGL1) and virus-heparan sulfate interactions without abolishing the interaction between the virus and scavenger receptor B2 (SCARB2). Sequencing of the plaque-purified RA-resistant viruses revealed a N104K mutation in the five-fold axis of the structural protein VP1, which contains positively charged amino acids reportedly associated with virus-PSGL1 and virus-heparan sulfate interactions via electrostatic attraction. The plasmid-derived recombinant virus harbouring this mutation was confirmed to be refractory to RA inhibition. Receptor pull-down showed that this non-positively charged VP1-N104 is critical for virus binding to heparan sulfate. As the VP1-N104 residue is conserved among different EV-A71 strains, RA may be useful for inhibiting EV-A71 infection, even for emergent virus variants. Our study provides insight into the molecular mechanism of virus-host interactions and identifies a promising new class of inhibitors based on its antiviral activity and broad spectrum effects against a range of EV-A71.

## Introduction

Enterovirus A71 (EV-A71 or EV71) is a nonenveloped single-stranded RNA virus with a positive-sense genome approximately 7400 bases in length. EV-A71 is human enterovirus of the family Picornaviridae. Its viral RNA is enclosed within a pentameric icosahedral capsid, which encodes a polyprotein that is processed into structural proteins (VP1∼VP4) and nonstructural proteins (2A∼2C and 3A∼3D) by viral proteases (2A^pro^, 3C^pro^, and 3CD^pro^) [1]. The capsid proteins VP1, VP2, and VP3 form the outer surface of the capsid, whereas VP4 forms the inner capsid. Studies have indicated that receptor binding triggers the uncoating process [2]. The externalization of VP4 and the N-terminus of VP1 results in release of the viral genome [3].

EV-A71 commonly causes hand, foot, and mouth disease in young children. Although patients with this disease are typically asymptomatic or mildly symptomatic, several outbreaks complicated by neurological diseases, such as myocarditis, acute flaccid paralysis, and encephalitis, or deaths, have been reported in the Asia-Pacific region since 1997 [4–8]. A very recent outbreak involved 74 children at Colorado, USA, in 2018 who presented with neurological manifestations [9]. However, other than symptomatic treatments, no treatment for EV-A71 infection is available. Thus, studies to develop anti-EV-A71 agents are urgently needed.

Viral virulence is determined by efficient infection, which relies heavily on efficient entry of the viral particles into the host cell and release of its genome for replication. Many cellular receptors, including the most studied scavenger receptor class B member 2 (SCARB2), P-selectin glycoprotein ligand-1 (PSGL1), and cell surface heparan sulfate glycosaminoglycan, have been reported [10]. Human SCARB2, a ubiquitously expressed protein that functions in membrane transport, was shown to function as an attachment receptor and an uncoating factor for EV-A71 entry [11]. SCARB2 interacts with the EV-A71 VP1 protein and triggers uncoating of EV-A71 under low pH conditions. The acidic environment causes a pH-dependent conformational alteration of VP1 and dislodges the “pocket factor” from the hydrophobic pocket beneath the canyon region of the virions, permitting initiation of the uncoating process [2]. X-ray structures revealed that the WIN compounds, such as DBPR103 and PR66, replace the natural pocket factor within the hydrophobic pocket, stabilizing the viral particle and preventing virus uncoating [12–14]. Moreover, PSGL1, a pan-selectin ligand, is expressed on leukocytes, platelets, and endothelial cells. Mouse cells overexpressing human PSGL1 are susceptible to EV-A71 [15]. However, unlike SCARB2, PSGL1 may not affect virus uncoating [11]. Cell surface heparan sulfate glycosaminoglycan is involved in the entry processes of many viruses [16]. For EV-A71, based on the structural model of the EV-A71 pentamer, the binding site for heparan sulfate is located around the symmetrical five-fold axis containing clustering of the positively charged amino acids, such as VP1-K242 and VP1-K244 [17]. Recently, the suramin derivative NF449 and tryptophan dendrimers were shown to interact with the five-fold vertex effectively blocking attachment of EV-A71 to PSGL1 and heparan sulfate [18,19]. Hence, EV-A71 can be classified as PSGL1-binding [20] and PSGL1-non-binding (non-PB) strains [20]. VP1-145 is located on the virus surface, surrounding the positively charged VP1-K242 and VP1-K244 surrounding the five-fold axis of symmetry. Exposure of the VP1-244K side chain, controlled by VP1-145, is critical for binding of EV-A71 to PSGL1 [21].

**Figure 1.**
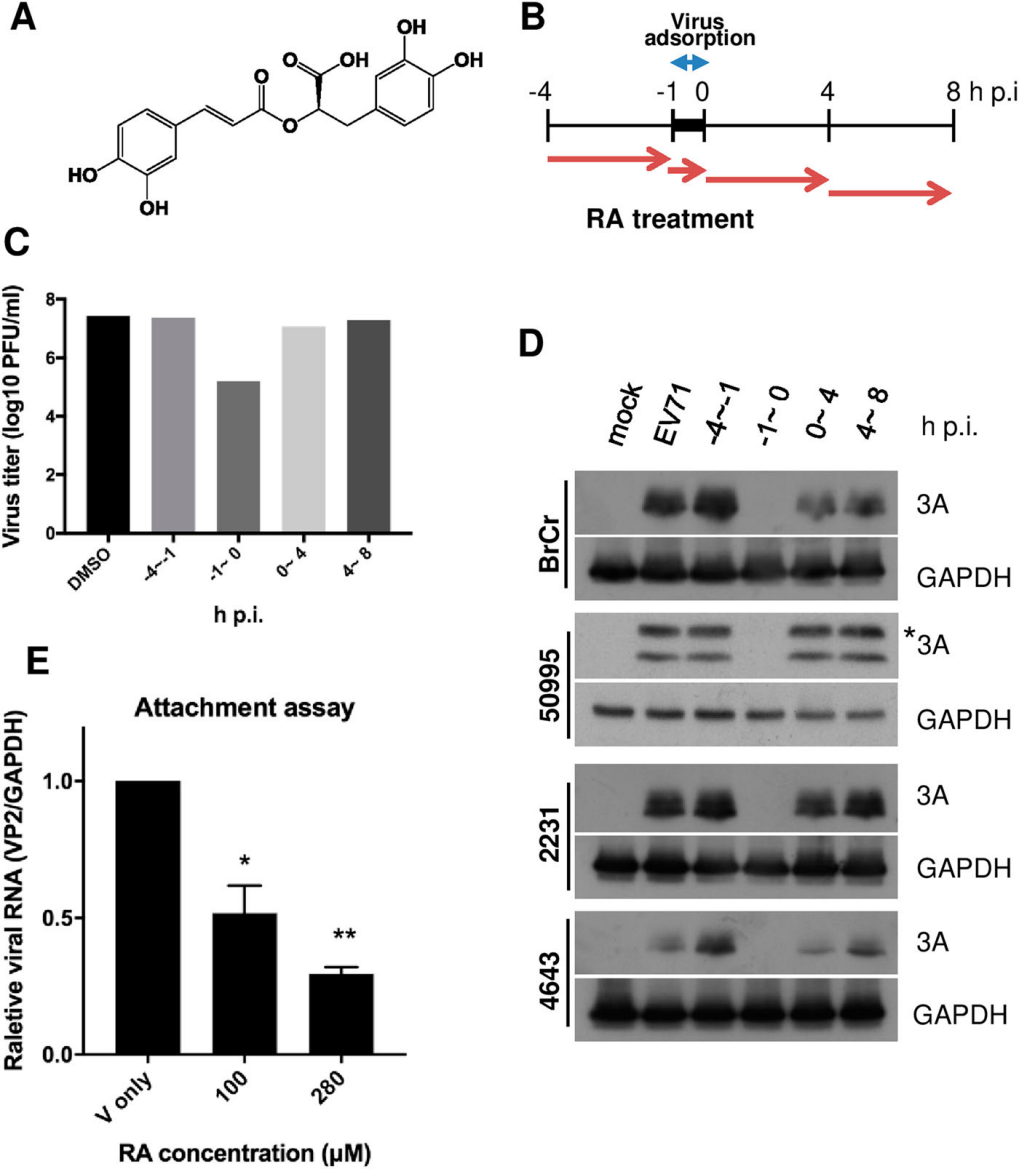
RA inhibits EV-A71 infection. (A) Chemical structure of RA. (B–D) Anti-EV-A71 activities of RA at different time points of addition. (B) RD cells were infected with the indicated viruses at a multiplicity of infection (MOI) of 10 in the presence of RA (280 μM, converted from 100 μg/mL) treated at the various time points: prior to infection (−4 – −1 h p.i.), during virus infection (−1–0 h p.i.), and after virus entry (0–4 h p.i. and 4–8 h p.i.). DMSO (0.1%) was used as the vehicle control. Viruses and cell lysates under each condition were collected at 8 h p.i. for plaque assay analysis (C) and western blot analysis (D), respectively. The data shown are representative of two independent experiments. (D) The EV-A71 strains were BrCr, TW/50995/12, TW/2231/98, and TW/4643/98. * denotes the 3AB intermediate. (E) Attachment assay. The viruses were incubated with DMSO or RA (280 μM) at 4°C for 1 h, and then infected into RD cells at 4°C for another 1 h. The amount of attached virus was estimated by quantitative real-time RT-PCR. The data are expressed as the mean ± SD from three independent experiments and analysed by the Student’s *t*-tests (*P < 0.05 and **P < 0.01).

Previously, during screening of the anti-EV-A71 activity of herbal extracts in a cell-based anti-cytopathic effect (CPE) assay, we observed that *Salvia miltiorrhiza* (Danshen) inhibits EV-A71 at viral entry (adsorption) [22]. Rosmarinic acid (RA), an ester of caffeic acid and 3,4-dihydroxyphenyllactic acid (Figure 1A) found in Danshen, exhibits antioxidant, anti-inflammatory, anti-bacterial, and antiviral properties [23–26]. RA has also been previously shown to possess anti-EV-A71 activity, as it inhibits virus entry [22,27]. However, the underlying molecular mechanism remains uncharacterized. In this study, we examined the mechanism by which RA blocks virus entry into the host.

## Materials and methods

### Cell culture and virus stock

Human rhabdomyosarcoma (RD) cells were obtained from the American Type Culture Collection (ATCC). L929 cells expressing PSGL1 (L-PSGL1 cells) were provided by Dr. Satoshi Koike of Tokyo Metropolitan Institute of Medical Science [11]. RD and L-PSGL1 cells were cultured at 37°C with 5% CO_2_ in Dulbecco’s Modified Eagle Medium (DMEM; Gibco BRL) supplemented with 10% (v/v) fetal bovine serum (FBS) (JRH Biosciences). Jurkat cells were provided by Dr. Ming-Ling Kuo of Chang Gung University and cultured at 37°C with 5% CO_2_ in RPMI 1640 medium (Gibco BRL) supplemented with 10% (v/v) FBS. EV-A71 strain 2231 (TW/2231/98) was derived from an infectious cDNA clone shared by Dr*.* Mei*-*Shang Ho of Academia Sinica. A mouse-adapted EV-A71 strain MP4 was provided by Dr. Jen-Ren Wang of National Cheng Kung University [28]. EVD68 US/MO/14-18947 and US/KY/14-18953 were from ATCC. EV-A71/5865/sin/000009 (subgenotype B4, GenBank accession no. AF316321) and its variants harbouring VP1-K242A and VP1-K244A all carried VP1-145Q [17]. The other viruses listed in Table 1 and Figure 1 were from the Clinical Virology Laboratory of Chang Gung Memorial Hospital, Taiwan. Virus propagation and titre determination using the plaque assay were performed in the RD cells [22].

**Table 1.** Inhibition spectrum of RA against different viruses.

Cell line or virus strain	CC_50_ (μM)^a^	EC_50_ (μM)^b^	SI^c^
RD cells^d^	327.68 ± 14.43		
A549 cells	216.82 ± 14.61		
EV-71/Taiwan/50995/12 (genotype B)		31.57 ± 4.14	10.36
EV-71/Taiwan/51045/12 (genotype B)		35.15 ± 1.21	9.30
EV-71/Taiwan/51126/12 (genotype B)		39.13 ± 2.51	8.36
EV-71/Taiwan/2557/12 (genotype B)		36.58 ± 1.42	8.94
EV-71/Taiwan/2231/98 (genotype C)		41.41 ± 0.45	7.90
EV-71/Taiwan/4643/98 (genotype C)		82.43 ± 0.79	3.97
EV-71/Taiwan/4643/MP4 (genotype C)		114 ± 4.10	2.87
Adenovirus		>100	
EV-D68 Taiwan/14-02795		>100	
EV-D68 US/KY/14-18953		>100	
EV-D68 US/MO/14-18947		>100	

^a^CC_50_: Drug concentration that caused 50% cytotoxicity. ^b^EC_50_: Concentration of compounds that inhibited cytopathic effects caused by 50% of the viruses. EC_50_ values are the mean  ±  SD of the results from two to three independent experiments. ^c^SI: CC_50_/EC_50_. ^d^RD cells were used for enteroviruses and A549 were used for adenovirus.

### Reagents and antibodies

RA was purchased from Sigma-Aldrich (St. Louis*,* MO, USA)*.* Mouse anti-GAPDH antibody was from Abnova (Taipei, Taiwan). Rabbit polyclonal antibodies against EV-A71 3A was prepared in house [29]. Mouse anti-3D antibody was a gift from Dr. Shin-Ru Shih of Chang Gung University. Mouse monoclonal [10F0] to EV-A71 was purchased from Abcam. Goat anti-SCARB2 and mouse anti-PSGL1 were purchased from Santa Cruz Biotechnology (Santa Cruz*,* CA). Protein G Mag Sepharose® Xtra was from GE Healthcare.

### Determination of cytotoxicity and virus-induced CPE of RA

The cytotoxicity of RA was determined by incubating RD cells with RA for three days, and the surviving cells were then stained with MTT (3-[4.5-dimethylthiazol-2-yl]-2,5-diphenyl tetrazolium bromide) [30]. The 50% cytotoxic concentration (CC_50_) was calculated as the RA concentration that reduced cell viability by 50%. For EC_50_ determination, RD cells were infected with 9TCID_50_ of each virus in the presence of the indicated concentrations of RA. After incubation at 37°C for 3 days, the cells were fixed and stained with crystal violet as described previously [30]. EC_50_ was defined as the concentration of RA that reduces the virus-induced CPE by 50%.

### Animal experiments

The EV-A71 mouse model was established as described previously [28]. All animal protocols were approved by the Institutional Animal Care and Use Committee of the Chang Gung University (CGU10-001). Briefly, 5-day-old specific pathogen-free ICR mice were purchased from Lasco (Taipei, Taiwan). RA was administered orally twice a day with the indicated dose (100 mg/kg/day) starting from day six of age. The pups were set apart from the mother for 8 h (starvation condition) to reduce the fullness of the stomach and prepare them for the intraperitoneal virus challenge on day seven of age (virus strain: MP4; 2 × median lethal dose: 1 × 10^6^ PFU in 100 µL). RA was prepared in a final volume of 100 μL in 5% DMSO aqueous solution; equal volume of 5% DMSO was used as the negative control. The infected mice were monitored daily for body weight, survival, and disease score. The disease scores were set as follows to represent the progression of virus infection: score 1, slow movement; score 2, one hind-limb paralysis; score 3, both hind-limb paralysis; score 4, death [14].

### Time-of-addition assay

RD cells (5 × 10^5^ cells/ well) were seeded in six-well plates for 24 h. The cells were then infected with various viruses at an multiplicity of infection (MOI) of 10 and incubated for 1 h at 4°C. The cells were washed with phosphate buffered saline (PBS) to remove the unbound virus and were maintained in DMEM supplemented with 2% FBS. RA (280 μM) was added at the indicated time points. Virus from the culture supernatant and cells in each condition were collected and pooled. The virus titre was assessed using the plaque assay and viral protein expression levels were monitored using western blotting.

### Attachment assay

RD cells were seeded in six-well plates for 24 h. The virus (5 × 10^6^ PFU) was preincubated with DMSO or RA (280 μM) at 4°C for 1 h. The cells were then incubated with the virus for 1 h at 4°C to allow virus binding but not internalization. The cells were washed with PBS to remove unbound virus and then dissolved in the TRIzol reagent. Total RNA was extracted and 1 µg total RNA was reverse-transcribed using the M-MLV reverse transcriptase system (Invitrogen). Quantitative PCR (qPCR) was performed using the StepOnePlus real-time (RT) PCR system (Applied Biosystems, Foster City, CA) with the following specific primer pairs: VP2 forward primer 5′- CTGATGGCTTCGAATTGCAA-3′ and reverse primer 5′- GCGTTTATGTACGGCACTATTATTGT-3′; GAPDH forward primer 5′-TGCACCACCAACTGCTTAGC-3′ and reverse primer 5′-GGCATGGACTGTGGTCATGAG-3′. The target genes were then amplified under the following conditions: 50°C for 2 min, 95°C for 10 min, 50 cycles at 95°C for 15 s, and 60°C for 1 min. To quantify the changes in viral RNA expression, the 2^−ΔΔCT^ method was used to calculate relative fold changes normalized to the GAPDH control.

### Centrifugal filtration assay

Viruses (10^5^ PFU) were incubated with DMSO or RA (280 μM) in a total volume of 1 mL at 4°C for 1 h. The mixtures were transferred to 100-kDa Amicon centrifugation filter units (Millipore) and centrifuged at 8000 × *g* for 20 min at 4°C. After washing twice with DMEM, the concentrated viruses were resuspended in DMEM and then subjected to plaque assay for titre determination.

### Generation and selection of RA-resistant viruses

Monolayers of RD cells seeded in 10-cm culture dish were infected with the EV-A71 strain 2231 at an MOI of 0.1 in the presence of RA (280 μM) or the DMSO control. When 90% of cell CPE was observed in the DMSO-treated group, the viruses (designated P1-RAr and P1-RAs, respectively) were collected and stored at −80°C. For each round of passaging, equal volume of P1-RAr or P1- RAs (500 μL) were used to infect fresh RD cells. The selections were terminated at passage 15, when no further decreased susceptibility to RA compared to passage 10 was obtained. Six plaques of P10-RAr and three plaques of P10-RAs were then isolated via plaque purification to further confirm the results of the resistance assay. After propagation in RD cells, the viral RNAs were extracted using TRIzol reagent (Invitrogen). One microgram RNA was reverse-transcribed using the SuperScript® II reverse transcriptase kit (Invitrogen) with random hexamers to generate first-strand cDNA. The cDNAs were used as templates to amplify the P1, P2, and P3 regions of the EV-A71 genome with the following primers: P1F: 5′-GCATGGCTAGCATGGGCTCCACGGTGTCCA-3′, P1R: 5′-CGAATGAATTCGTGAGAGTGGTAATTGCTGTG-3′, P2F: 5′-GAAGGCACAACCAACCCGAAAGGGTACG-3′, P2R: 5′-ATCGATGAATTCGTTTGAAAACCGGCGAACAAC-3′, P3F: 5′-GCATGGCTAGCATGGGACCCCCTAAATTTAG-3′, and P3R: 5′-CGACTGAATTCGTAAACAATTCGAGCCAATTTC-3′. The amplified RT–PCR products were ligated into the TA cloning vector (T & A cloning kit, Yeastern Biotech, Taiwan) and sequenced to determine the mutation sites.

### EV-A71- receptor binding inhibition assay

RA or DMSO was incubated with EV-A71 (20 µg) in 1 mL of 5% FBS-DMEM at 4°C for 1 h. The mixture was further incubated with Protein G Mag Sepharose® Xtra (GE Healthcare) conjugated with the extracellular fragment of SCARB2-hFc (1 μg, R & D Systems) or PSGL1-hFc (1 μg, R & D Systems) at 4°C for 2 h. The beads were washed twice with DMEM and then mixed with sodium dodecyl sulfate-polyacrylamide gel electrophoresis (SDS-PAGE) sample buffer. The precipitated SCARB2-hFc, PSGL1-hFc, and viruses were analysed using western blotting with the indicated antibodies. For the heparan sulfate pull-down assay, RA or DMSO was incubated with EV-A71 in 1 mL PBS at 4°C for 1 h. Then, the mixture was incubated with heparan sulfate-Sepharose 6 Fast Flow (GE Healthcare Life Sciences) at 4°C for another 2 h. After washing twice with PBS, the beads were suspended in SDS sample buffer and subjected to SDS-PAGE and immunoblotting with EV-A71 antibody. For pulldown of the wild-type 5865/sin/000009 strain and its variant VP1-K242A and VP1-K244A viruses, equal RNA copy numbers in 50 µL of sequence-verified, passage P1 viruses from cDNA clones were used in a heparan sulfate pulldown assay [17]. Precipitated viruses were quantified via RT-qPCR against a standard viral RNA curve using specific primers [17,30].

### Growth curve of viruses

RD cells were grown in 6-well plates and infected with the plasmid-derived viruses at a MOI of 10 for various times post-infection (p.i.). The progeny viruses were collected at the indicated time points for titre determination using the plaque-forming assay.

### Statistical analysis

Data are expressed as the mean ± standard deviation and were analysed using the two-tailed Student’s *t*-tests. *P *< 0.05 was considered statistically significant.

## Results

### RA affects the early stage of virus infection

We assessed the efficiency of RA against various EV-A71 strains and other DNA and RNA viruses. As shown in Table 1, RA selectively inhibited all tested EV-A71 of both genotypes B and C, whereas no inhibitory activity against enteroviruses D68 was observed. Additionally, no antiviral activity was detected against adenovirus with DNA as the genomic material (Table 1). As target discovery is critical for drug development and application, we characterized the mode of action of RA by performing a time-of-addition assay. RD cells were treated with RA for the times indicated in Figure 1B. Viruses under each condition were collected at 8 h p.i., and the viral titres were determined in a plaque assay (Figure 1C). The results indicated that the virus titre was most prominently reduced when RA was added at the time of virus infection (–1–0 h p.i.) (Figure 1C). Further, the inhibitory effect of 280 µM RA was not cytotoxic (data not shown). To further confirm the results in different strains of EV-A71, time-of-addition studies were performed using immunoblot analysis to monitor the expression level of viral nonstructural protein 3A. The presence of RA was required at the time of virus infection to observe an antiviral effect (Figure 1D). In addition, an attachment assay was conducted to evaluate the binding of EV-A71 to RD cells in the presence of RA. Consistently, the amount of bound virus was significantly suppressed by RA treatment (Figure 1E). Together, these results indicate that RA acts at the early stage of EV-A71 infection.

### RA may exert its antiviral effects by directly targeting EV-A71

**Figure 2.**
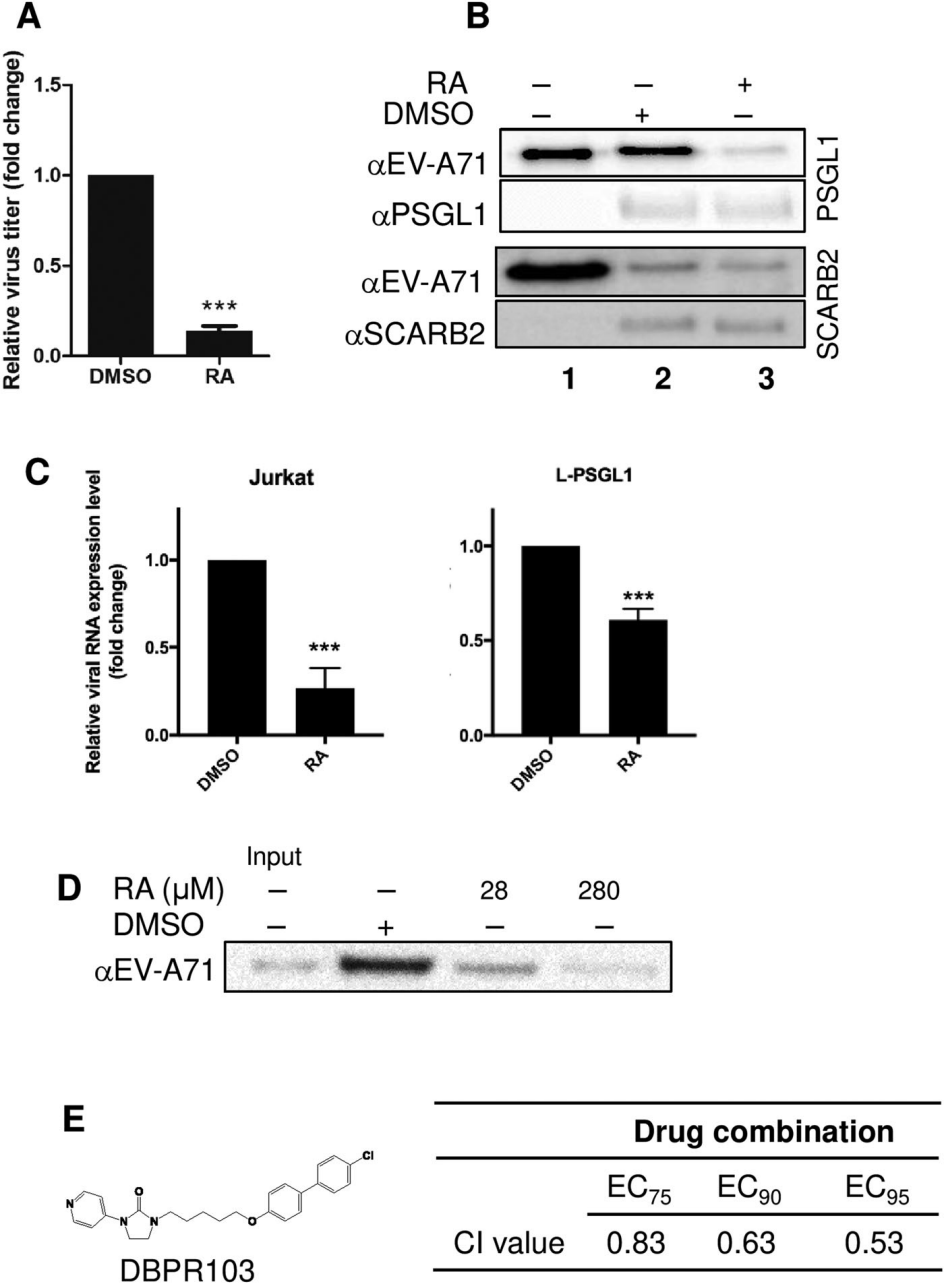
RA exerts its antiviral effects by directly targeting virus-host receptor interaction. (A) Centrifugal filtration assay. The data are expressed as the mean ± SD from three independent experiments and analysed by Student’s *t*-tests (***P < 0.001). (B) Effect of RA on the binding of EV-A71 to SCARB2 and PSGL1. EV-A71 was pretreated with DMSO or RA (280 μM) at 4°C for 1 h and then incubated with protein G Mag Sepharose conjugated to extracellular fragments of PSGL1 (upper panels) and SCARB2 (lower panels). Lane 1 contains 0.2 µg virus samples as an input control. The results are representative of at least three independent experiments. (C) Attachment assay using L-PSGL1 cells and Jurkat cells. Viruses were incubated with DMSO or RA (280 μM) at 4°C for 1 h and then infected into cells at 4°C for another 1 h. The amount of the attached virus was estimated by real-time RT-PCR. The data are expressed as the mean ± SD from three independent experiments and analysed by Student's *t*-test (***P < 0.001). (D) Effect of RA (28 or 280 μM) on binding of EV-A71 to heparan sulfate beads. The 2231/1998 virus was pre-treated with RA before binding to the heparan sulfate beads, and the precipitated viruses were evaluated through western blotting. The results are representative of at least three independent experiments. (E) Effect of the combination of RA and DBPR103 on anti-EV-A71. (Left) Chemical structure of DBPR103. (Right) The CI values for drug combination. CI > 1, CI = 1, and CI < 1 indicated antagonistic, additive, and synergistic effects, respectively.

As the results of time-of-addition and virus attachment assays indicated that RA targets the virus or the cellular receptor(s), we performed a centrifugal filtration assay to further examine the antiviral effect of RA. Compared to the DMSO-treated control, RA-treated virus showed an approximately 90% reduction in the virus titre (Figure 2A), indicating that RA directly targets the virus. We next evaluated whether RA interferes with the interaction between the virus and host receptor during virus infection. We used receptor pull-down assays to detect interactions between the virus and known EV-A71 receptors, such as hSCARB2, hPSGL1, or heparan sulfate, in the presence of RA. We observed that RA treatment inhibited the binding of EV-A71 to PSGL1, but not SCARB2, as detected by western blotting with anti-EV-A71 antibodies (lane 2 versus lane 3 of Figure 2B). The results of an attachment assay in Jurkat T cells constitutively expressing PSGL1 and mouse L929 cells ectopically expressing human PSGL1 (L-PSGL1) [15] confirmed that RA inhibited the interaction of EV-A71 and PSGL1 (Figure 2C). Accordingly, both PSGL1 and heparan sulfate may interact directly with the positively charged residues near the five-fold axis of the EV-A71 pentamer; thus, we investigated the effect of RA on virus-heparan sulfate binding. In a heparan bead pull-down assay, RA dose-dependently inhibited virus binding, as indicated by the decreased detection of viral proteins (Figure 2D). We performed a drug combination experiment to determine whether the combination of RA and DBPR103, a WIN compound that inhibits viral uncoating [13], synergized the inhibitory effect against EV-A71 (Figure 2E). The combination drug dosing was performed using fixed ratios of the EC_50_ values (20,000:1). Cell viability was examined after 24 h of treatment using the MTT assay. As shown in Figure 2E, enhancement (synergy) of the protective effect was observed when the combination index was ≤ 1 [31], indicating that the mode of action of RA may differ from that of DBPR103 as expected. Together, these results suggest that RA directly targets EV-A71, and thus interferes with the virus-PSGL1 or virus-heparan sulfate interaction.

### Selection, identification, and characterization of RA resistance

We isolated RA-resistant viruses to identify the possible RA target site. Full viral genomic sequencing of six plaque-purified viruses showed a reproducible neutral amino acid asparagine to positively charged lysine mutation at residue 104 (N104K) of VP1 (5 of 6 clones), indicating that the VP1 protein is a target of RA (Table 2). Identical mutations were not detected in the P2 and P3 regions among these six resistant clones. Particularly, one plaque-purified virus harbouring a glutamate to glycine substitution at residue 98 (E98G) of VP1 was also isolated through selection. Both VP1 N104 and E98 are within the BC loop of VP1, a region exposed on the viral surface that may participate in virulence determination. We determined whether VP1 N104K and E98G were resistance-conferring mutations. To this end, we assessed the drug susceptibility of recombinant viruses bearing single amino acid mutations (N104K variant and E98G variant) derived from the cDNA clone. The inhibitory effect of RA on the N104K variants was undetectable; however, the E98G variant remained sensitive to RA treatment, indicating that N104K, not the E98G mutation, was responsible for the inhibitory effect of RA (Table 3). The resistance of E98G identified during selection may have arisen from the combination of other mutations in VP1. A comparison of growth curves for the wild-type and the mutant viruses revealed that the N104K mutation in VP1 (in the absence of RA) negligibly affected the replication kinetics, indicating that the N104K mutation affected the fitness of the virus (Figure 3A). Furthermore, the results of the attachment assay demonstrated that RA treatment did not affect infectivity of the N104K variant compared to that of the wild-type virus (Figure 3B). Taken together, these results indicate that the N104K mutation is an important determinant of RA resistance.Figure 3.VP1 mutation confers resistance to the inhibitory effects of RA. (A) Growth curves of WT and N104K viruses. The data shown is from one of two independent experiments. (B) Attachment of wild-type and N104K variant viruses with RD cells. The amount of the attached virus was estimated using quantitative RT-PCR and was normalized the DMSO-treated control, set as 1. The data were expressed as mean ± SD from three independent experiments and analysed using the Student's *t*-test (*P < 0.05; **P < 0.01). (C-D) Effect of RA on the binding of EV-A71 variants to heparan sulfate and PSGL1-hFc. The EV-A71 variants were first pretreated with DMSO or RA at 4°C for 1 h and were then incubated with heparan sepharose beads (C) or PSGL1-hFc-conjugated protein G Mag Sepharose (D). The results are representative of at least three independent experiments. In (D), the ratio of EV-A71 binding to PSGL1 was defined by the levels of EV-A71 proteins over PSGL1. (E–F) Effect of RA (28 or 280 μM) on binding of non-heparan sulfate binding viruses to heparan sulfate beads or RD cells. (E) Equal copy numbers of viruses were subjected to heparan sulfate binding analysis. Bound viruses were assessed using RT-qPCR. (F) Viruses were incubated with RD cells in the presence of DMSO or RA on ice for 1 h. The medium was replaced with E2 containing RA and incubated for another 6 h. Total RNA was harvested using TRIzol for RT-qPCR. RNA copy number of each experiment was normalized to wild-type (left panel) or respective DMSO control (right panel), arbitrarily set as 1. The data are expressed as the mean ± SD of three independent experiments and were analysed by Student's *t*-test. *P < 0.05, **P < 0.01, and ns, no significance.

**Figure:**
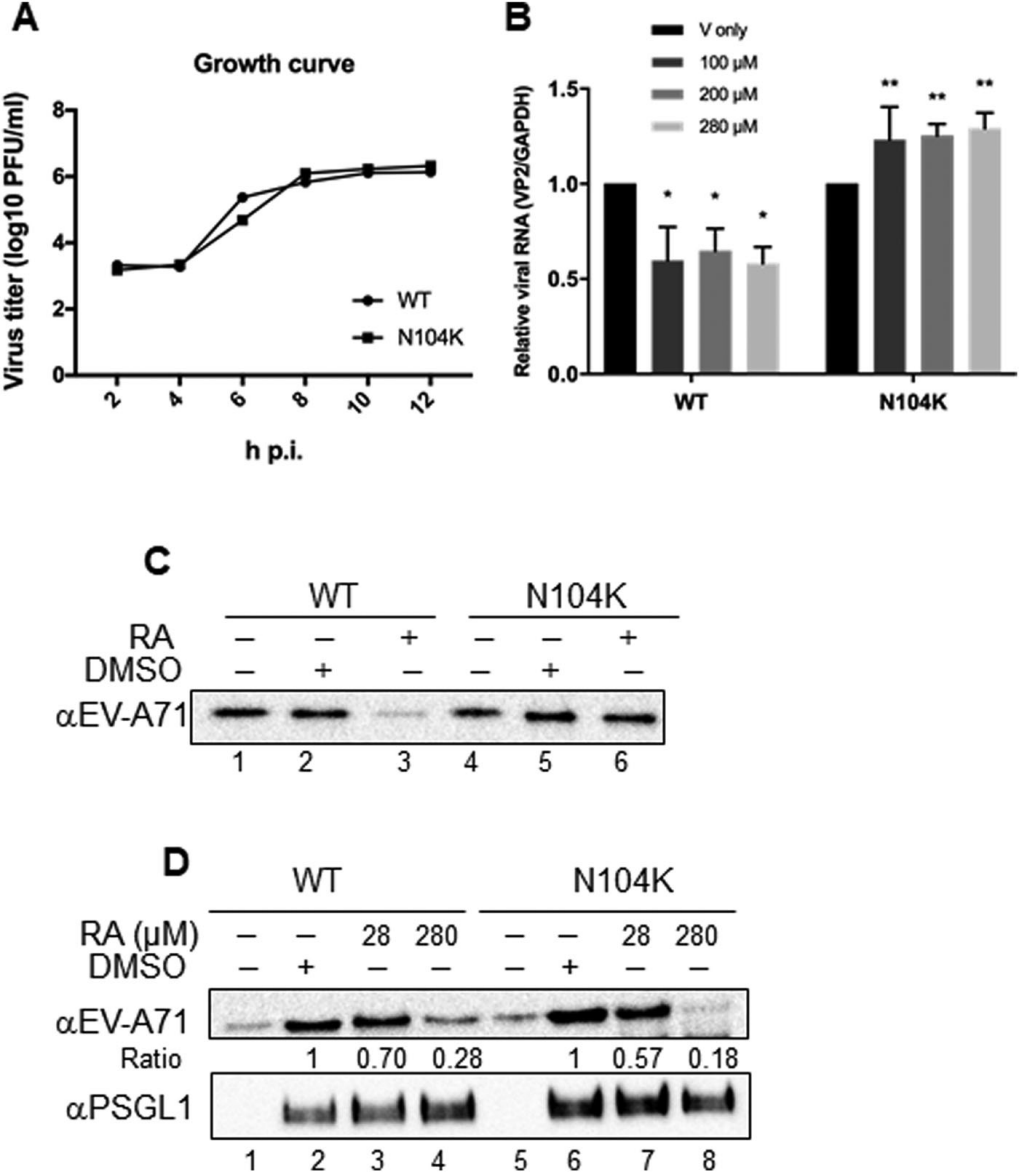

**Figure:**
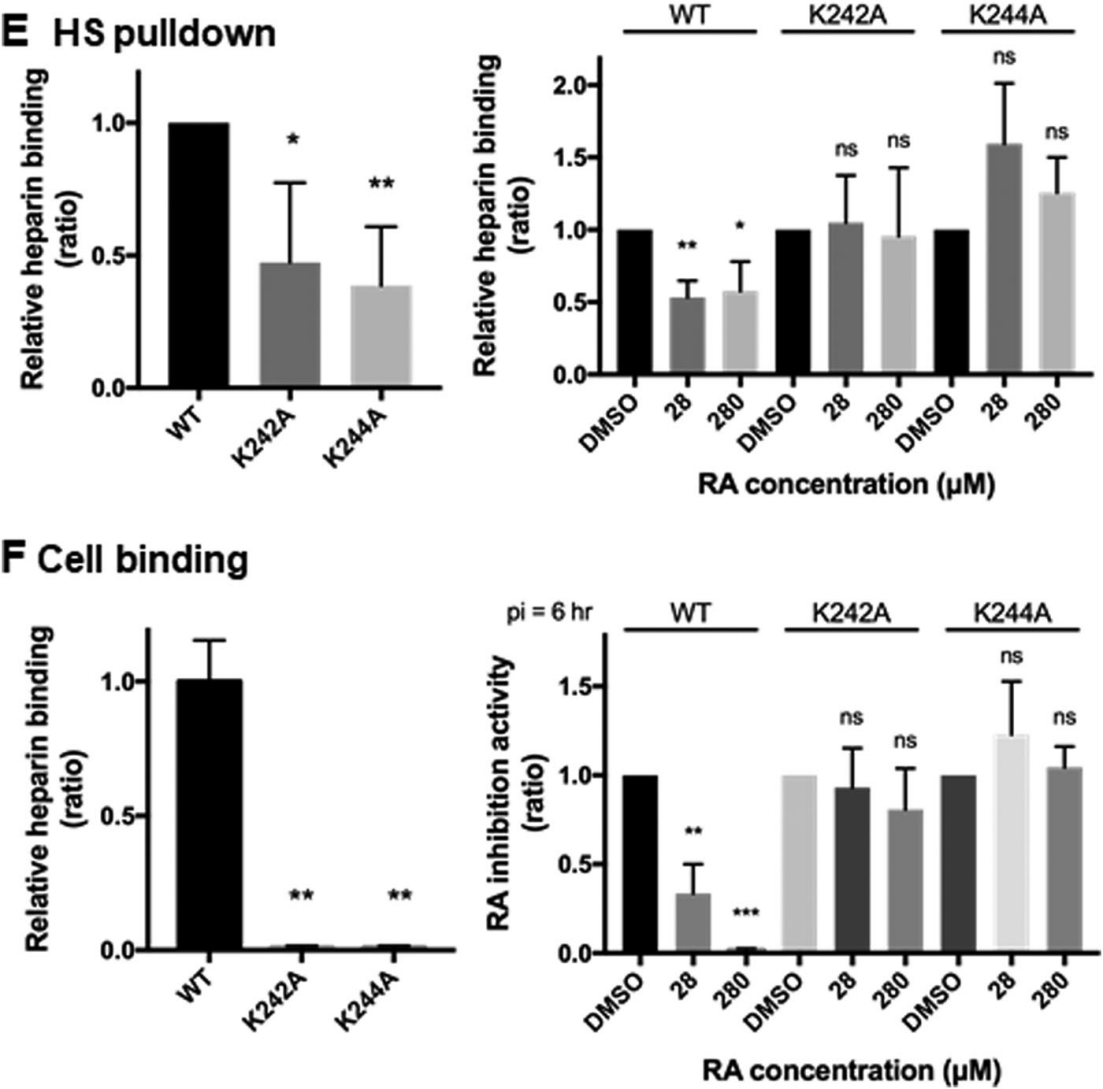

**Table 2.** Complete-genome sequencing of WT and RA-resistant viruses isolated from plaque purification.

Virus	Amino Acid substitution		
	P1	P2	P3
Control virus-**1**			3C V157A
Control virus-**2**		2B G19S	3A P45H
		2C G132V	
Control virus-**3**			3B V15A
			3C K55R
RA R-**1**	**VP1 N104K**		
RA R-**2**	**VP1 N104K**	2C L125V	3A I78V
	VP2 C41Y		3D R49K
			3D I269L
RA R-**3**	**VP1 N104K**	2A G49S	
	VP4 E21G		
RA R-**4**	**VP1 N104K**	2B K98R	3D P202T
	VP1 N108S	2C D294G	
	VP2 D57N		
	VP3 K79E		
RA R-**5**	**VP1 E98G**		3C E182D
	VP1 F131C		
	VP1 H257R		
	VP2 K149M		
RA R-**6**	**VP1 N104K**	2C D252G	3D S173P
	VP2 G227S

**Table 3.** Anti-EV-A71 activities of cDNA-derived recombinant viruses harbouring the E98G or N104K mutations in VP1.

	EC_50_ (μM)
EV-A71 TW/2231/1998 (wildtype)	41.41 ± 0.45
EV-A71 TW/2231/1998 (E98G)	44.95 ± 2.22
EV-A71 TW/2231/1998 (N104K)	>100

### Receptor pull-down showed that asparagine at position 104 in VP1 is critical for virus binding to heparan sulfate but not to PSGL1

Next, we identified the mutation responsible for the reduced sensitivity to virus-heparan sulfate interaction in the presence of RA (Figure 3C). Interestingly, the N104K mutation generated a positively charged lysine residue, which did not alter virus binding to the heparan sulfate receptor in the absence of RA (lane 5, Figure 3C). The results of the heparan sulfate bead pull-down assay showed that RA did not inhibit the interaction between the N104K variant and heparan sulfate, although the amount of wild-type virus was low in the heparan sulfate bead precipitates after RA treatment (lane 3 versus lane 6, Figure 3C). However, inhibition of the virus-PSGL1 interaction was not alleviated by the N104K mutation in the receptor pull-down assay (Figure 3D). Thus, the N104K mutation may account for the resistance to virus-heparan sulfate but not the virus-PSGL1 interaction. As previously reported [17,18], non-heparan sulfate binding viruses, harbouring VP1-K242A and VP1-K244A, displayed reduced binding with heparan sulfate beads in DMSO-treated controls compared to the wild-type virus, 5865/sin/000009 (left panel; Figure 3E). Binding of wild-type virus to heparan sulfate beads was susceptible to inhibition by RA, but the K242A and K244A variants were not significantly inhibited at up to 280 μM of RA (right panel, Figure 3E). Similarly, these mutant viruses showed little binding to RD cells, as reflected by their low RNA synthesis (left panel, Figure 3F). These viruses were also resistant to inhibition by RA by binding to the cell surface, indicating specific inhibition of RA (Figure 3F).

### Proposed docking pose of RA with EV-A71 VP1 protein

**Figure 4.**
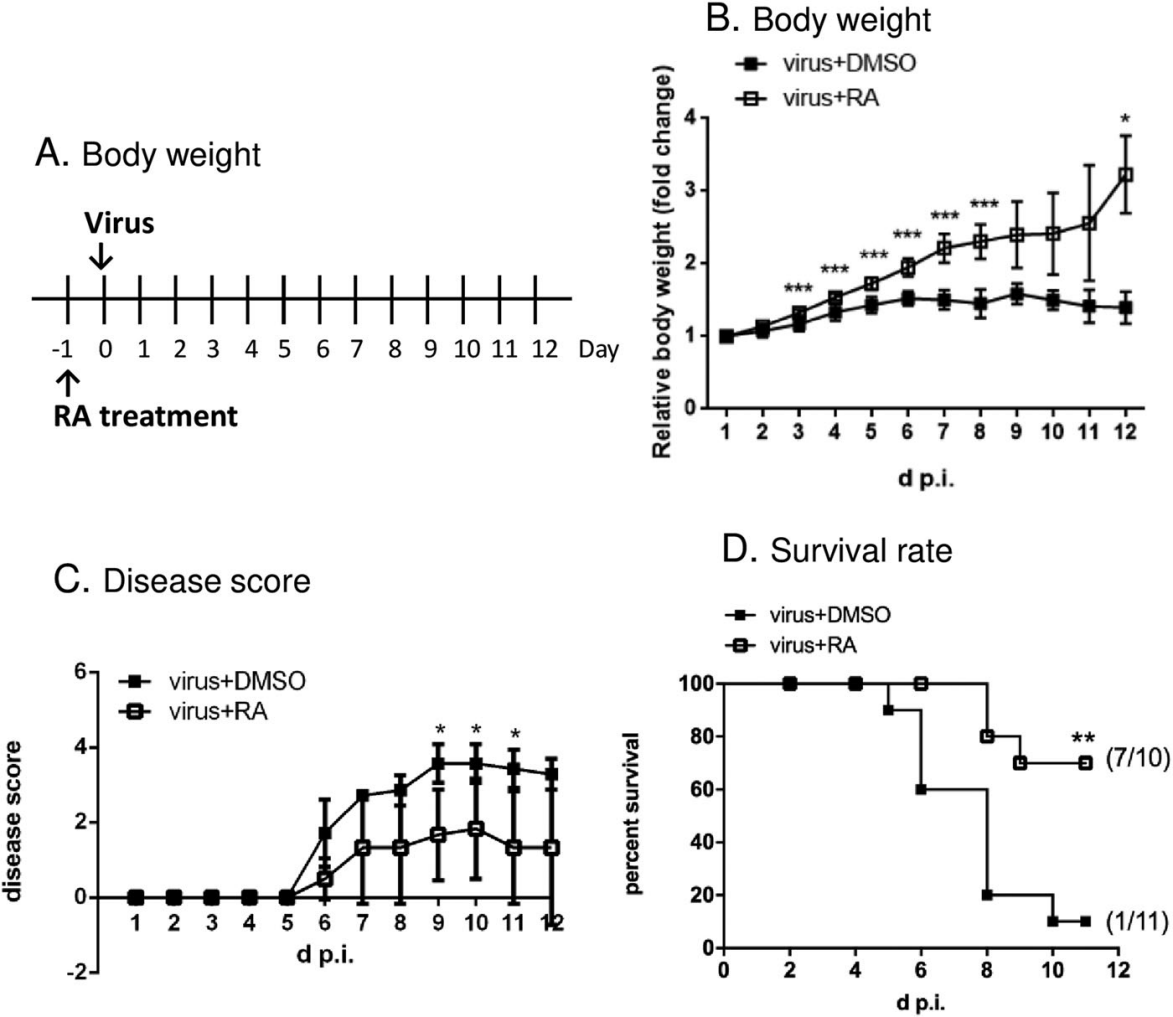
Role of RA in protection against the EV-A71 challenge in vivo. (A) An illustration of the treatment with RA in an animal model. (B) Effects of RA on body weight loss of infected mice. Values show the mean ± SD; *n* = 6. (C) Effects of RA on the relief of symptoms of infected mice. Values show the mean ± SD; DMSO group, *n* = 7 and RA group, *n* = 6. (D) Effects of RA on survival of infected mice. The mice survival was estimated using the Kaplan-Meier method; DMSO: *n* = 11, RA: *n* = 10. *P < 0.05, **P < 0.01, and ***P < 0.001.

As the VP1 N104 residue may be the target of RA, we docked RA onto the constraint area, which encompasses this residue. The results revealed a potential binding site surrounding the cluster of E98, K242, and K244 in the PB model (Figure S1): RA interacted with N104, K242, S243, and P96 of the wild-type VP1 protein. RA also bound to K104 via hydrogen bonds in the docking model of N104K VP1 protein. The docking position of RA, however, is distant from the five-fold axis. We further analysed the VP1 sequence of different EV-A71 viruses, which have been classified into genotypes A, B, and C based on the VP1 sequence. The results showed that N104 is highly conserved (Table S1), suggesting that RA has broad targeting potential against EV-A71 infection, which was confirmed in an inhibition spectrum assay (Table 1). To verify this proof of concept, we determined whether RA inhibited EV-A71 in an animal model. We treated mice with control vehicle or RA orally one day before the infection of the mouse-adapted strain MP4 (Figure 4A). We observed that the vehicle-treated mice exhibited a flattened weight curve following viral infection, whereas the body weight of RA-treated mice significantly increased by approximately 3-fold after 12 days of administration (Figure 4B). The vehicle-treated mice showed paralysis at day 7 post-infection, and most died within 12 d p.i. RA treatment ameliorated the severe neurological symptoms (Figure 4C). Furthermore, treatment of the mice with RA clearly reduced mortality following infection; the survival rate was 70% after 11 days of observation (Figure 4D). Overall, these results demonstrated the protective role of RA against EV-A71 infection both *in vitro* and *in vivo*.

## Discussion

Here, we tested the known compounds of Danshen to evaluate their bioactivity against EV-A71. Danshen extract targets the viral entry stage and exhibits high inhibitory specificity, as it exclusively inhibits strains of EV-A71 not those of other enteroviruses or DNA viruses [22]. Using a chemical genetic approach, we identified RA as the potential active ingredient in Danshen, as it specifically targeted the viral entry step similar to that of Danshen (Figure 1 and Table 1). RA may exert its antiviral activity by directly targeting the viral particles, as the result of the centrifugal filtration assay showed that virus pretreated with RA inhibited viral infection (Figure 2A). This was also supported by the identification of RA-resistant viruses harbouring the N104K mutation in VP1 (Table 2). Residue 104 is positioned in the five-fold symmetric vertex containing positively charged amino acid residues K244, K242, and R166, which have been reported to be involved in the virus-heparan sulfate interaction via electrostatic interactions with the negatively charged sulfate receptor [17,21]. Mutations of these individual positively charged amino acids severely affected heparan sulfate binding [17]. In addition, Tan et al. identified the N104S variant in a non-heparan sulfate-binding population through next-generation sequencing [17]. Substitution of VP1-N104 with a lysine residue enhanced the EV-A71-heparan sulfate interactions as VP1-104 is close to VP1-K242 and K244 (residues responsible for heparan sulfate binding). This may impact the RA-EV-A71 interaction, as observed in the resistant virus selection experiment. Interestingly, the N104K mutation generated a positively charged lysine residue, which did not alter virus binding to the heparan sulfate receptor in the absence of RA (lane 5, Figure 3C). Our results from the pull-down assay showed that RA negatively affected virus binding to PSGL1 and heparan sulfate (Figure 2). Using the same pull-down approach, we also ruled out the possibility that RA affects the virus-hSCARB2 interaction (Figure 2). hSCARB2 binds to the canyon region composed of the VP1 GH and VP2 EF loops, which is distinct from the five-fold apex of the VP1 where the N104 is in the BC loop [32].

**Figure 5.**
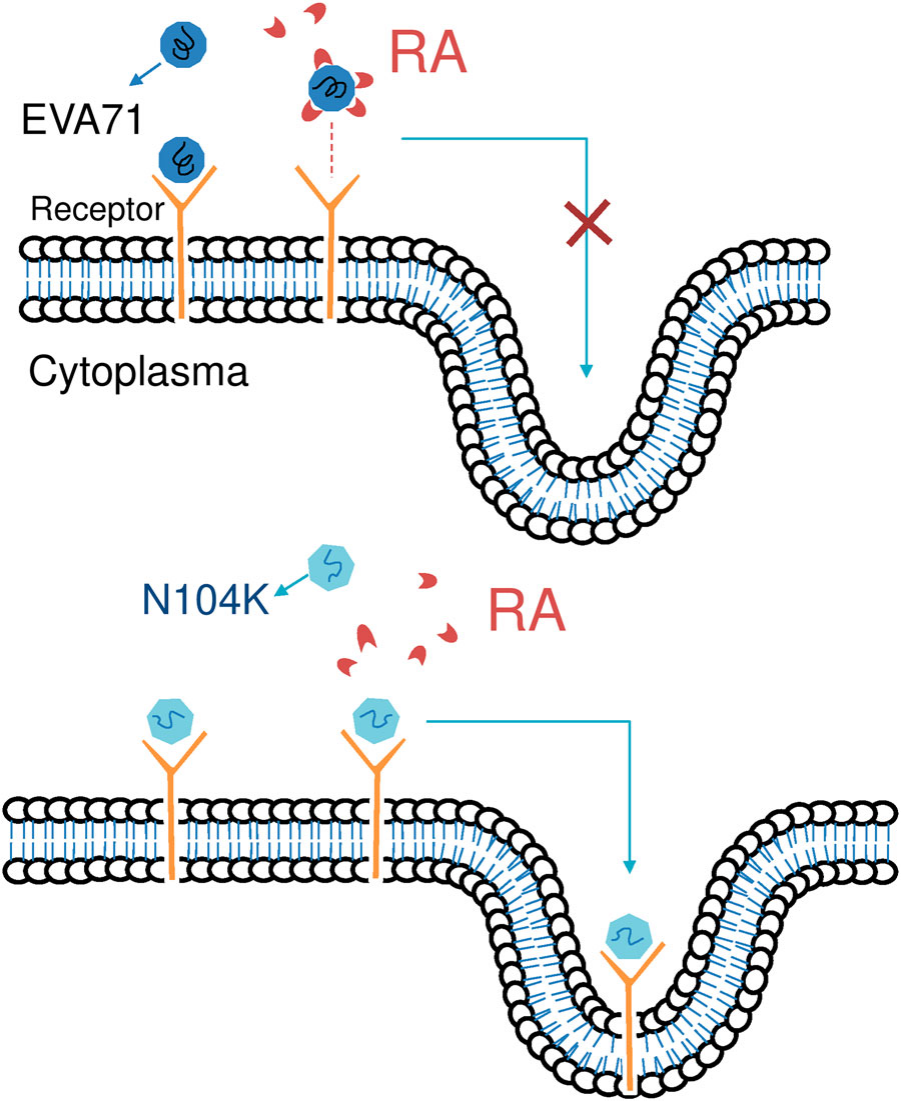
Hypothetical model showing the effect of RA on EV-A71’s entry in host cells. (A) RA binds to EV-A71, preventing the virus from binding to the receptor and inhibiting its entry into the host cell. (B) We proposed that the amount of RA binding to the recombinant mutant virus will be less due to the specific N104K mutation, and the virus entry will not inhibited.

The cell surface heparan sulfate functions as a receptor for numerous viruses because of the presence of positively charged amino acid residues in the five-fold axis [16]. However, RA did not inhibit adenovirus infection (Table 1), a representative DNA virus, which also uses cell surface heparan sulfate as the entry receptor [33], indicating that RA specifically targets EV-A71. RA displayed the distinct ability to affect the growth of the genetically similar EV-A71 and EV-D68, which have similar molecular mechanisms of viral replication. The virion of EV-D68 also possesses typical five-fold axis structures and uses heparan sulfate as a receptor [34], but was not inhibited by 100 µM RA (Table 1). This indicated that RA does not target cellular proteins, common to EV-A71 and EV-D68 replication. This was supported by the observation that RA did not target host cells, as viral replication was not inhibited when RA was preincubated with RD cells before virus adsorption in the time-of-addition assay (Figure 1). We thus proposed a hypothetical model showing the effect of RA on EV-A71 entry into host cells (Figure 5).

PSGL1 is expressed on immune cells and thus may be associated with virus-induced severe complications in patients with hand, foot, and mouth disease [35]. Our data showed that RA inhibited EV-A71 binding to PSGL1 in the receptor pull-down assay, although the amino acid responsible for this binding was not identified in the resistant virus selection assay (Table 2). RD cells are non-immune cells derived from muscle rhabdomyosarcoma and expresses negligible levels of PSGL1 as shown by RT–PCR analysis [36], and thus no resistant virus against PSGL1 was selected. We postulated that the VP1-145 was responsible for the resistance, as a variant virus of EV-A71/Taiwan/2231/98 harbouring 145E (non-PB) instead 145Q [20] in VP1 became resistant to RA in a virus-induced CPE assay (data not shown). In addition, VP1-145 has been shown to control virus binding to PSGL1 [21]. The effects of RA on events associated with PSGL1 binding using immune cells warrant further investigation in the future.

Recently, virus strains with intra- or inter-typic recombination have been reported [37]. Recombination events may alter virulence and result in epidemics. The results of multiple sequence alignments indicated that the highly conserved VP1 N104 residue is present among various EV-A71 strains (Table S1). Therefore, RA may be useful for treating EV-A71 infection, even for emergent virus variants. Our findings indicate that RA is a multiple-target drug against EV-A71-PSGL1 and -heparan sulfate. Although RA inhibited EV-A71 infection with EC_50_ values in the micromolar range, our observations regarding the mechanism of action of RA require further investigation to develop more potent derivatives or combinational therapies.

## Supplementary Material

Supplemental Material
