# Supplementary material for: Rosmarinic acid exhibits broad anti-enterovirus A71 activity by inhibiting the interaction between the five-fold axis of capsid VP1 and cognate sulfated receptors
Source: Emerg Microbes Infect. 2020 Jun 4;9(1):1194–205. doi: 10.1080/22221751.2020.1767512 (PMC7448925; doi:10.1080/22221751.2020.1767512)
Supplement: Supplemental Material [file TEMI_A_1767512_SM6068.zip › 1767512_Suppl files/Supplemental Information.docx]

## Supplemental Information

## Simulation of the molecular docking of EV-A71 VP1 and RA

The structural interaction between VP1 and RA was analysed using the molecular modelling software Discovery Studio 4.1 (Acers, CA, USA). The EV-A71 crystal structure (PDB ID: 4AED) was obtained from the RCSB Protein Data Bank. The three-dimensional structure of RA was generated by the Chem 3D ultra 12.0 software (Cambridge Soft Corporation, MA, USA). The initial structures were prepared using the CHARMM force field and minimised. A sphere radius of 30 Å was set to assign the surface binding site involving three loops, P96-G105, D164-L169, and V238-Y245. Different chemical conformations of RA were generated and docked between RA and VP1 following the CDOCKER protocol in Discovery Studio 4.1. The random conformations were set to 200, and other parameters were set to the software defaults.

**Figure S1. The putative docking pose of RA with the EV-A71 VP1 protein.** The three-dimensional structure of the EV-A71 VP1 five-fold region (PDB 4AED) was used in the molecular docking studies. The putative binding conformation and the corresponding interactions between RA and VP1 were identified. The orange and green dotted lines indicate hydrogen bonds, and the pink dotted line represents charge interaction.

**Table S1. Amino acid sequence alignment of the EV-A71 VP1 protein of the indicated strains.**
