# Supplementary material for: Rosmarinic acid exhibits broad anti-enterovirus A71 activity by inhibiting the interaction between the five-fold axis of capsid VP1 and cognate sulfated receptors
Source: Emerg Microbes Infect. 2020 Jun 4;9(1):1194–205. doi: 10.1080/22221751.2020.1767512 (PMC7448925; doi:10.1080/22221751.2020.1767512)
Supplement: Supplemental Material [file TEMI_A_1767512_SM6068.zip › 1767512_Suppl files/Table_S1._Amino_acid_sequence_alignment_of_the_EV_A71_VP1_protein_of_various_genotypes.docx]

**Table S1.** Amino acid sequence alignment of the EV-A71 VP1 protein of various genotypes.

| **Strain**  **(Genotype)** | **Amino acid sequence**  (90)........98^a^....104......(110) (141).......145^a^.....(150) | |
| --- | --- | --- |
| **4643/98** | (90)VGEIDLPL E GTTNPNGYANWD(110) | (141)TP T G E VVPQL(150) |
| **EV-A71/BrCr** | (90)........ K .....N......(110) | (141).. . . R .....(150) |
| **Genotype B0** | (90)........ K .....N......(110) | (141).. . . Q .....(150) |
| **Genotype B1** | (90)........K/E.....N......(110) | (141)..S/T.E/G/Q.....(150) |
| **Genotype B2** | (90)........ E .....N......(110) | (141).. . . E/G .....(150) |
| **Genotype B3** | (90)........ E .....N......(110) | (141).. . . E/G .....(150) |
| **Genotype B4** | (90)........ E .....N......(110) | (141).. . .E/G/Q.....(150) |
| **Genotype B5** | (90)........E/K.....N......(110) | (141).. . .E/Q/G.....(150) |
| **Genotype C1** | (90)........ E .....N......(110) | (141).. . . E .....(150) |
| **Genotype C2** | (90)........ E .....N......(110) | (141)..T/S. E/Q .....(150) |
| **Genotype C3** | (90)........ E .....N......(110) | (141).. . . E .....(150) |
| **Genotype C4** | (90)....... E/K.....N......(110) | (141).. . .E/Q/G.....(150) |
| **Genotype C5** | (90)........ E .....N......(110) | (141).. . . E .....(150) |

^a^Order according to the probability of each amino acid.
