# Supplementary figures and images for: Rosmarinic acid exhibits broad anti-enterovirus A71 activity by inhibiting the interaction between the five-fold axis of capsid VP1 and cognate sulfated receptors
Source: Emerg Microbes Infect. 2020 Jun 4;9(1):1194–205. doi: 10.1080/22221751.2020.1767512 (PMC7448925; doi:10.1080/22221751.2020.1767512)

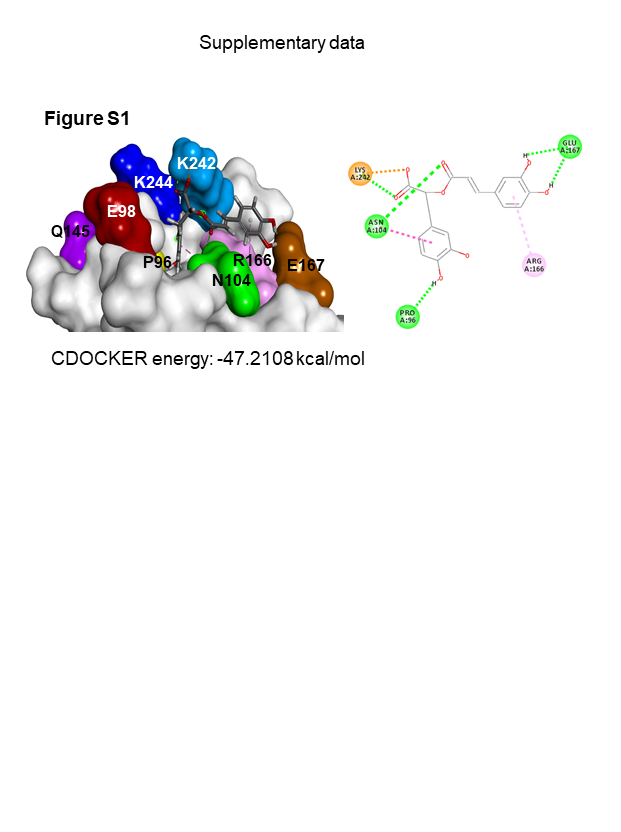

Supplement: Supplemental Material [file TEMI_A_1767512_SM6068.zip › 1767512_Suppl files/Figure_S1_final.TIF]
